# Recent Advances in Our Understanding of HLA-G Biology: Lessons from a Wide Spectrum of Human Diseases

**DOI:** 10.1155/2016/4326495

**Published:** 2016-08-29

**Authors:** Fabio Morandi, Roberta Rizzo, Enrico Fainardi, Nathalie Rouas-Freiss, Vito Pistoia

**Affiliations:** ^1^Laboratory of Oncology, Istituto Giannina Gaslini, 16147 Genoa, Italy; ^2^Department of Experimental and Diagnostic Medicine, Section of Microbiology, University of Ferrara, 44121 Ferrara, Italy; ^3^Neuroradiology Unit, Department of Diagnostic Imaging, Azienda Ospedaliero-Universitaria Careggi, 50134 Florence, Italy; ^4^CEA, Institut des Maladies Emergentes et des Therapies Innovantes (iMETI), Service de Recherche en Hemato-Immunologie (SRHI), Hôpital Saint-Louis, 75010 Paris, France

## Abstract

HLA-G is a HLA-class Ib molecule with potent immunomodulatory activities, which is expressed in physiological conditions, where modulation of the immune response is required to avoid allograft recognition (i.e., maternal-fetal interface or transplanted patients). However, HLA-G can be expressed* de novo* at high levels in several pathological conditions, including solid and hematological tumors and during microbial or viral infections, leading to the impairment of the immune response against tumor cells or pathogens, respectively. On the other hand, the loss of HLA-G mediated control of the immune responses may lead to the onset of autoimmune/inflammatory diseases, caused by an uncontrolled activation of the immune effector cells. Here, we have reviewed novel findings on HLA-G functions in different physiological and pathological settings, which have been published in the last two years. These studies further confirmed the important role of this molecule in the modulation of the immune system.

## 1. Introduction

HLA-G is an important molecule with immunomodulatory properties, which belongs to ‘‘nonclassical” HLA-class Ib molecules, along with HLA-E, -F, and -H [1]. In contrast with ‘‘classical” HLA-class Ia molecules (HLA-A, -B, and -C), which are highly polymorphic, with a high number of alleles encoding a high number of functional proteins, HLA-Ib molecules display a very low polymorphism, with a small number of alleles encoding a limited number of proteins. Similarly to HLA-Ia molecules, also HLA-Ib molecules can bind peptides generated through a proteasome-driven degradation of cytosolic proteins and present them to specific subpopulations of CD8^+^ T cells [2]. However, the main function of these molecules is to modulate the immune responses in both physiological and pathological conditions [3].

HLA-G molecule can be present in seven different isoforms encoded through alternative splicing of the same primary transcript. Membrane-bound isoforms (HLA-G1, -G2, -G3, and -G4) lack intron-4, which is retained in the soluble (s) isoforms (HLA-G5, -G6, and -G7), leading to the splicing of the transmembrane domain. In addition, sHLA-G1, which is structurally identical to sHLA-G5, can be generated by shedding of membrane-bound HLA-G1 isoform through metalloproteases-mediated cleavage [4]. Only HLA-G1 and -G5 present a full length heavy chain, which is associated with *β*2-microglobulin and binds small peptides, although it is unclear whether or not HLA-G can present them to CD8^+^ T lymphocytes [5]. In this respect, Diehl et al. have characterized specific motifs in HLA-G restricted peptides and they have concluded that HLA-G can present peptides similarly to classical HLA-class I molecules [6].

HLA-G can interact with immunoglobulin-like transcript 2 (ILT2), which is expressed by T and B lymphocytes, Natural Killer (NK) cells, monocytes/macrophages, and dendritic cells, and with ILT4, which is expressed only by myeloid cells (i.e., dendritic cells, monocytes, and macrophages and neutrophils) [7]. In addition, HLA-G can interact with KIR2DL4 expressed by NK cells [8] and CD160 expressed by T lymphocytes, NK cells, and endothelial cells [4]. Upon interaction with these receptors, HLA-G can affect the function of different cell populations. In particular, HLA-G (i) impairs T cell function, by inhibiting proliferation [9] and cytotoxicity [10] and by inducing apoptosis [11] and expansion of regulatory T cells [12, 13], (ii) inhibits differentiation, proliferation, and cytokine production in B lymphocytes [14], (iii) inhibits proliferation and cytotoxicity [15, 16] of peripheral blood NK cells and induces proliferation and release of proangiogenic factors in blood and uterine NK cells [17–19], (iv) inhibits chemotaxis of different T, B, and NK cell populations by downregulating chemokine receptors expression on their surface [20], (v) inhibits phagocytosis and production of reactive oxygen species in neutrophils [7], and (vi) dampens angiogenesis, by inhibiting endothelial cells proliferation [21].

In the last years, several papers have demonstrated that HLA-G can be present as a homodimeric molecule, which is generated through a disulfide bond [22–24]. Moreover, it has been observed that homodimer is more biologically active than the monomeric form [13, 25, 26].

## 2. HLA-G and Cancer

Overexpression of membrane-bound soluble and sHLA-G has been detected in different human solid and hematological tumors and might represent a mechanism performed by tumor cells to escape from the control of the immune system, by inhibiting NK and T cells mediated lysis [4].

In the last two years, several papers have addressed the role of HLA-G in tumor progression or have characterized this molecule as prognostic factor for the clinical outcome of cancer patients (Table 1).

Loumagne et al. have performed an interesting study using a murine model. In fact, HLA-G can interact with murine paired immunoglobulin-like receptor- (PIR-) B, ortholog of human ILT receptors, thus enabling the investigation of its role* in vivo*. Immunocompetent mice were injected either with syngeneic tumor cells coexpressing human HLA-G5 and *β*2-microglubulin (h*β*2m) or with h*β*2m^+^ HLA-G5^−^ tumor cells. Interestingly, h*β*2m^+^ HLA-G5^−^ tumors were rejected, whereas h*β*2m^+^ HLA-G5^+^ tumors secreted soluble HLA-G, which protected them from h*β*2m-elicited immune rejection, and grew similarly to a poorly immunogenic tumor. They demonstrated that HLA-G5 tumor expression dampened anti-h*β*2m B cell response through accumulation of myeloid-derived suppressor cells which inhibited T cell proliferation and reduced T and B cell tumor infiltrate [27].

Zheng et al. have demonstrated that HLA-G (mostly HLA-G1 and -G5) is expressed by tumor lesions in esophageal squamous cell carcinoma (ESCC) patients, but not in adjacent normal tissues or in healthy controls. Moreover, such expression positively correlated with lymph node metastasis and cancer cell differentiation. Accordingly, sHLA-G serum levels were higher in patients than in controls [28].

An interesting study has been performed by Reimers and colleagues on rectal cancer patients. They analyzed tumors by tissue microarray for the presence of Foxp3^+^ cells (Tregs) and tumor expression of HLA-E and HLA-G. They observed that patients with rectal tumors characterized by loss of HLA class I expression, Foxp3^+^ infiltration below median, and weak HLA-G expression displayed a worse overall survival (OS) and disease-free survival (DFS) [29]. In contrast, Guo et al. have demonstrated that the majority of colorectal cancer tissues tested positive for HLA-G or HLA-E expression, and half of them expressed both molecules. Moreover, expression levels of HLA-G or HLA-E and the combined expression of both molecules were all negatively correlated with OS of colorectal cancer patients. However, these authors found that only HLA-G expression can serve as independent factor for OS, whereas the expression of HLA-E was significantly correlated with tumor metastasis [30]. Yan et al. have analyzed the expression of soluble HLA-G5/G6 molecules in tumor samples from non-small-cell lung cancer (NSCLC) patients, using specific mAb 5A6G7. sHLA-G expression was observed in half of NSCLC lesions and was significantly higher in adenocarcinoma lesions than that in squamous cell carcinoma and adenosquamous carcinoma lesions. The authors concluded that sHLA-G could be a useful biomarker to discriminate adenocarcinoma from squamous cell carcinoma in NSCLC patients [31].

Another interesting study comes from Xu and colleagues. They analyzed HLA-G expression in a cohort of patients with pancreatic carcinoma and found high levels of HLA-G in the majority of tumor samples. Moreover, they demonstrated that HLA-G levels represented an independent predictor for patients' OS, and a positive correlation was found between HLA-G expression and tumor stage, extrapancreatic infiltration, lymph node involvement, and poor differentiation. Finally, they demonstrated that plasma levels of sHLA-G were higher in patients than in healthy controls, and sHLA-G levels inversely correlated to numbers of peripheral activated T cells, thus suggesting that sHLA-G promotes tumor immune escape through the inactivation T cell responses [32].

A study performed by Wang et al. described a relationship between HLA-G expression and the sharpness of low-grade glioma tumor borders in magnetic resonance images. In particular, high HLA-G expression was detected in larger tumors with blurred boundaries, which may be those prone to diffuse invasion. Therefore, patients with tumors that highly expressed HLA-G were less likely to have undergone complete resections [33].

Expression and function of HLA-G and regulatory microRNA (miR-152, -148A, -148B, and -133A) have been analyzed by Jasinski-Bergner and colleagues in renal cell carcinoma (RCC). They have observed an inverse correlation between the expression of miR-148A and -133A and HLA-G protein* in situ* and* in vitro*. They concluded that a stable miRNA overexpression downregulated HLA-G expression, thus enhancing NK and LAK mediated cytotoxicity. This hypothesis has been confirmed by the analysis of immune cell infiltration [82]. They have also identified two additional miRNAs (miR-548q and miR-628-5p) which may regulate HLA-G expression in RCC, demonstrating a direct interaction of these miRNAs with the 3′ untranslated region (3′ UTR) of HLA-G. Stable overexpression of miR-548q and miR-628-5p caused a downregulation of HLA-G mRNA and protein, leading to an enhanced NK cell-mediated cytotoxicity. In addition, they found an inverse correlation between the expression of miR-628-5p and HLA-G protein in primary RCC lesions and cell lines. The authors concluded that these miRNAs, which are able to tune HLA-G expression, might serve as future therapeutic targets [34]. The interactions between HLA-G and miR-152 (and TGF-*β*) have been also analyzed by Guan and colleagues in gastric cancer (GC). They observed a positive correlation between serum levels of TGF-*β* and HLA-G in GC patients and a direct TGF-*β*-mediated induction of HLA-G expression in GC cell lines* in vitro*. Furthermore, TGF-*β* also inhibited miR-152 expression, and HLA-G was posttranscriptionally regulated by miR-152. In addition, miR-152 overexpression repressed HLA-G upregulation induced by TGF-*β*. Finally, the authors observed that miR-152 expression levels were inversely correlated to both HLA-G and TGF-*β* levels in GC patients. They suggested a potential application of miR-152 as therapeutic target or potential biomarker for GC patients [35]. Also the study carried out by Xu et al. has focused on HLA-G expression in GC. The authors demonstrated that (i) human tolerogenic dendritic cells DC-10, which express HLA-G, are dramatically increased in the peripheral blood of GC patients as compared to healthy donors, (ii) the expression of HLA-G on these cells is significantly higher than in DC-10 cells from healthy donors, and (iii) concentration of plasma sHLA-G is higher in GC patients than controls. Finally, they demonstrated that these three parameters are worse prognostic factors for GC patients, thus suggesting an immunosuppressive role for HLA-G and DC-10 cells in GC [36].

A novel interesting study has been published by Wu et al., who have characterized the antitumor potential of NK cells genetically modified to downregulate the expression of LILRB1. They have demonstrated that LILRB1^−^ NK cells proliferate upon stimulation signals, migrate, and eliminate HLA-G^−^ targets cells as parental NK cells do. Moreover, LILRB1^−^ NK cells exhibit higher proliferation, conjugate formation, degranulation, and killing activities compared to parent NK cells in the presence of HLA-G^+^ target cells. Finally, LILRB1 silencing rescued NK cell antitumor activity in a xenograft cancer model [37].

The effect of HLA-G expression by tumor cells on tumor infiltrating lymphocytes (TIL) has been also addressed by Zhou et al. in pancreatic cancer (PC). They detected HLA-G overexpression in tumor samples from PC patients, but not in nontumor tissues. The number of CD3^+^ TIL was significantly lower in HLA-G^+^ than in HLA-G^−^ tumors. More importantly, the authors demonstrated that HLA-G expression and low intratumoral CD3^+^ staining represent independent prognostic factors pointing to worse OS of PC patients. These findings suggest that HLA-G expression impairs host antitumor immune response and predicts a poor prognosis in PC [38].

König and colleagues have demonstrated for the first time that HLA-G might also serve as prognostic marker to predict the clinical outcome of neoadjuvant chemotherapy (NACT). In fact, they analyzed total sHLA-G and HLA-G levels in extracellular vesicles (EV) in plasma samples from breast cancer (BC) patients, before and after NACT. They found that total and free sHLA-G levels were higher in treated patients than in healthy controls. More importantly, high sHLA-G in EV before NACT positively correlated to disease progression, whereas free sHLA-G levels were directly correlated to a better clinical outcome, thus demonstrating that different sHLA-G subcomponents may have different prognostic impacts on the clinical outcome of NACT treated BC patients [39]. Zidi et al. have also focused on HLA-G function in BC patients. They analyzed sHLA-G in plasma samples obtained from women with BC and correlated sHLA-G concentration with pregnancy and breastfeeding history. They reported significant differences in sHLA-G levels between BC patients with/without breastfeeding experience. Interestingly, BC patients without previous pregnancy experience display higher levels of sHLA-G, and patients without both pregnancy and breastfeeding history showed a significant enhancement in tumor size compared with patients who had both experiences. These data suggested that a previous pregnancy and breastfeeding experience may protect against advanced BC stages through reduced levels of sHLA-G [40].

Five novel and interesting studies have addressed the correlation of different polymorphism of* HLA-G* gene with tumor progression. Bielska et al. have investigated the influence of two HLA-G polymorphisms, HLA-G-725C/G/T and HLA-G 14-base pair, on the susceptibility to diffuse large B-cell lymphoma (DLBCL) and on the clinical course of the disease. They observed that frequencies of HLA-G-725GG or HLA-G-725GC genotype were lower, and those of the HLA-G ins/ins genotype were higher in the patients compared with controls. Moreover, patients carrying the HLA-G-725CC genotype presented a better OS than subjects with other HLA-G-725C/G/T polymorphisms. Patients with homozygous HLA-G del/del had a worse OS than subjects carrying the HLA-G del/ins or the HLA-G ins/ins genotype. On the basis of genotype distribution, authors defined two HLA-G genotype-based risk groups; a high-risk (HR) group presented a worse OS than low-risk (LR) patients [41]. Wiśniewski et al. have characterized several polymorphisms, three in* HLA-G* gene (-964A>G, -725C>G>T, and -716T>G in the promoter and a 14 bp ins/del in the 3′UTR), five in* LILRB1* gene (-5651G>A in intron 14, 5717C>T L622L, 5724G>A E625K, 5774 C>A P641P in exon 15, and 5806C>T in 3′UTR), and 9620 9A/10A polymorphisms in exon 7 of* KIR2DL* gene in NSCLC patients. They have observed that only one single nucleotide polymorphism (SNP) in* HLA-G* (-964A>G) and one in* LILRB1* (5724G>A) positively correlated with a higher risk of NSCLC. In addition, 5724G>A in* LILRB1* gene was associated with protection from tumor cell infiltration of regional lymph nodes. Finally, tumor tissue samples tested positive for HLA-G and LILRB1 protein expression, and those expression levels significantly correlated with tumor stage [42].

Zambra et al. have evaluated the impact of eight polymorphisms in the 3′UTR region on susceptibility to the development of prostate cancer (PCa). They identified the UTR-4 haplotype as a risk factor to PCa. Furthermore, the “non-14 bp Ins_+3142G_+3187A” haplotype, the +3003CT genotype, and the +3003C allele are also related to a higher susceptibility to PCa. They conclude that HLA-G 3′UTR polymorphism has a great influence on PCa susceptibility [43].

Khorrami and colleagues investigated whether HLA-G polymorphisms might serve as a potential risk factor for clinical outcomes in gastric adenocarcinoma (GAC). They found that G^*∗*^01:01:03:01 and G^*∗*^01:01:08 allele distributions are significantly higher in healthy controls than in GAC patients and seem to have protective effect. Moreover, the G^*∗*^01:01:03:01/G^*∗*^01:04:01 and G^*∗*^01:01:02:01/G^*∗*^01:01:08 genotypes frequencies are higher in healthy controls than in patients. They conclude that* HLA-G* gene polymorphisms could affect GAC induction and its outcome [83].

In this line, Rizzo and coauthors showed the prognostic value of the 14-base pair deletion (del) polymorphism (rs66554220) in the 3′ untranslated region of HLA-G, with del/del patients showing reduced overall survival of patients with chronic lymphocytic leukemia, as compared to those with other genotypes [45].

In summary, novel findings in the last two years confirmed that the expression of HLA-G is generally detected in tumors but not in normal tissues, and such expression positively correlated with tumor progression, metastasis, invasiveness, and worse prognosis of patients. The role of HLA-G (through the interaction with LILRB1) in the control of antitumor immune response has also been confirmed both in cancer patients and in murine models of human tumors. Finally, several polymorphisms of* HLA-G* and* LILRB1* genes have been characterized in the last two years, with positive or negative correlation with tumor stage and susceptibility of patients to develop tumors of patients' survival, thus confirming the important role of HLA-G in tumorigenesis and tumor progression.

## 3. HLA-G and Pregnancy

The important role of HLA-G in human pregnancy has been fully characterized in the last years. Different HLA-G isoforms are expressed by trophoblast cells at maternal-fetal interface. HLA-G expressed and released by trophoblast cells can interact with cellular receptors expressed by immune (T cells, NK cells, macrophages, and dendritic cells) and nonimmune (endothelial cells) cells present in the decidua, triggering either inhibitory or activating signals. Such interactions allow us to (i) limit maternal immune response against semiallogeneic fetal tissues by impairment of decidual NK cell cytotoxicity, T and B cell proliferation, and induction of apoptosis of activated CD8^+^ T cells, (ii) stimulate placental development through secretion of proangiogenic factors by decidual (d) NK cells and macrophages, and (iii) provide a protective effect for pregnancy outcome by stimulating IL-4 secretion by CD4^+^ T cells [84]. In this view, some novel interesting data have been obtained by Tilburgs and coworkers, who have isolated and characterized invading HLA-G^+^ extravillous trophoblasts (EVT) cells and HLA-G^−^ villous trophoblasts (VT). Using microarray and functional gene set enrichment analysis, they revealed a striking immune-activating potential for EVT, which was absent in VT. Moreover, they performed cocultures of HLA-G^+^ EVT cells with matched decidual immune cells, and they found that EVT induced differentiation of CD4^+^ T cells in CD4^+^CD25^hi^FOXP3^+^CD45RA^+^ regulatory T cells (Treg) and increased the expression of FOXP3 in these cells. Moreover, EVT did not enhance cytokine secretion in dNK (whereas stimulation of dNK with other* stimuli* confirmed the distinct cytokine secretion profiles of dNK compared to peripheral blood NK cells) [13]. The interaction between dNK and EVT at the maternal-fetal interface was further characterized in a second study, where the authors demonstrated that dNK interacts with filamentous projections from EVT enriched in HLA-G, which may represent the initial stage of synapse formation. Moreover, dNK in this area expressed HLA-G on the surface or in the cytoplasm. Activation of dNK downregulated HLA-G expression and restored cytotoxicity, whereas HLA-G expression was reacquired by incubation with EVT. These data suggested that HLA-G undergoes a cycle of trogocytosis, endocytosis, degradation, and finally reacquisition on dNK, upon interaction with EVT. However, dNK can be activated by cytokines and/or viral products to acquire the ability to control virus infection at the interface (i.e., in the presence of human cytomegalo virus-infected decidual stromal cells) [46]. Collectively, all these data confirmed that HLA-G^+^ EVT cells have unique properties in maternal-fetal tolerance establishment.

Immune cells in the decidual compartment have been further characterized by Djurisic et al., who have analyzed sHLA-G and cells from placental bed and peripheral blood in first trimester. They found an increased number of T cells with low CD4 or CD8 expression in the placental bed. Soluble HLA-G was increased in “uterine blood” as compared to peripheral blood. Moreover, KIR2DL4 and LILRB1 expression was upregulated on uterine NK cells. Finally, a correlation was found between uterine sHLA-G and the fraction of KIR2DL4+ uterine NK cells. The authors hypothesize that the phenotype of uterine NK cells may be influenced by HLA-G on trophoblast cells and by sHLA-G in the uterus [47].

Ferreira and coworkers have performed an interesting study to analyze the genetic regulation of HLA-G expression in the placenta. They found a novel cis-regulatory enhancer element 12 kb upstream of* HLA-G* gene (Enhancer L). More importantly, deletion of this enhancer ablated HLA-G expression in JEG3 cells and in primary human trophoblast cells isolated from placenta. They demonstrated that this enhancer is cell-type specific and contains motifs for transcription factors of CEBP and GATA (which are essential for placentation), thus suggesting that these factors may also control HLA-G expression on trophoblast [48].

In the last two years, some studies have addressed interesting novel aspects of the role of HLA-G in pregnancy (Table 2). Klitkou et al. have performed the first large study simultaneously measuring sHLA-G in both maternal and umbilical cord blood to test whether there is a correlation between sHLA-G levels in maternal and fetal blood. Therefore, they have measured the levels of sHLA-G1/-G5 in maternal blood and paired umbilical cord blood samples from gestational week 20 (GW20) and at term. Soluble HLA-G levels were significantly lower in maternal blood at term than in GW20. At term, sHLA-G levels were significantly higher in maternal blood than in umbilical blood. They conclude that sHLA-G is not freely transferred over the placental barrier. Finally, they found a correlation between HLA-G levels in maternal blood in GW20 and at term and between HLA-G levels in maternal blood and umbilical cord blood at term. This might be due to shared genetic factors which affect sHLA-G production in mother and child, or it may support the theory that sHLA-G in the pregnant woman and the fetus is partly derived from the placenta, which is a shared organ between mother and child [49]. In this line, Dahl et al. have correlated* HLA-G* polymorphisms in the 3′UTR to sHLA-G levels in maternal blood plasma samples from GW20 and at term, as well as in fetal umbilical cord blood samples. They have observed that higher numbers of 14 bp ins alleles in the fetus were associated with higher maternal sHLA-G levels at term (restricting the analysis to 14 bp ins/del heterozygous mothers). Furthermore, they found that increasing numbers of fetal 14InsG alleles are related to significantly increased levels of sHLA-G in maternal blood plasma samples at term in heterozygous 14DelC/14InsG mothers. They conclude that combined fetomaternal HLA-G genotypes are related to sHLA-G levels in maternal blood plasma [50].

It has been previously demonstrated that human seminal plasma contains HLA-G [85]. In a novel study, Dahl et al. found that sHLA-G levels in seminal plasma samples correlated with* HLA-G* 14 bp ins/del genotype of the men, with the* del/del* genotype showing the highest level and the* ins/ins* genotype showing the lowest level of sHLA-G. Higher seminal plasma levels of sHLA-G were found in couples where the female partner became pregnant after assisted reproduction treatments (ART), compared with couples where no pregnancy was achieved, thus suggesting a possible role of seminal sHLA-G as an immunomodulatory factor in the female reproductive tract before and at the time of conception [51]. The role of HLA-G in the success of* in vitro* fertilization (IVF) has been also addressed by Lashley et al., who have performed genotyping of women with recurrent implantation failure (RIF) and their partners for HLA class I, HLA class II, HLA-G, and KIR alleles. Results were compared with those obtained from couples with successful embryo implantation after their first IVF and normal fertile couples. They found a higher frequency of HLA-C2 and 14 bp insertion in HLA-G in women with RIF than in controls, and they conclude that these two genetic features represent a risk factor which may affect the success of IVF [52]. In this view, Rizzo and coworkers have demonstrated that sHLA-G levels in uterine flushing samples were lower in primary infertile women than in women with secondary infertility. In addition, a lower number of endometrial CD56^+^KIR2DL4^+^ NK cells were found in primary infertile than in secondary infertile women [53].

Two additional studies addressed the impact of HLA-G polymorphisms on the outcome of pregnancy. Quach and colleagues have addressed the correlation between SNP in the 3′UTR of the* HLA-G* gene and an increased risk of preeclampsia. They found that preeclamptic cases were associated with a G/G-genotype at SNP +3187. In addition, one SNP combination (+3027C/C and +3187G/G) was significantly more prevalent in preeclampsia cases. They hypothesized that HLA-G 3′UTR SNP-pair associations, and not individual SNPs, could be useful to predict susceptibility to preeclampsia [54]. In contrast, Agrawal et al. have addressed the correlation between SNP in the HLA-G 5′-upstream regulatory region (URR) and recurrent spontaneous abortion (RSA). They genotyped women with idiopathic RSA (and their partners) and control couples, and they found an increased risk for idiopathic RSA in women with mutant genotypes of -1179G>A, -725C>G/T, and -86A>C SNP. They observed a 3.5-fold increased risk for -1179G>A and 4.3-fold increased risk for -725C>G/T SNP among carriers of mutant parental genotypes in couples who have experienced idiopathic RSA. Finally, they demonstrated a downregulation of HLA-G expression in cases of idiopathic RSA with -1179G>A and -725C>G/T SNPs. They conclude that these SNP may affect pregnancy outcome through HLA-G downregulation [55].

Two interesting studies have been carried out by Beneventi and coworkers on gestational diseases. First, they analyzed plasma levels of pregnancy-associated plasma protein A (PAPP-A) and sHLA-G in women with gestational diabetes mellitus (GDM). They found that women with GDM had significantly lower first-trimester PAPP-A and sHLA-G concentrations than controls. In addition, sHLA-G levels increased during gestation in diabetic women, showing an opposite trend with respect to the controls. They conclude that PAPP-A and sHLA-G represent independent markers of GDM, and their variations may help to early unravel the onset of GDM [56]. In the second study, they analyzed sHLA-G levels in plasma and cord blood samples from pregnant women with preexisting rheumatic diseases and unaffected pregnant women. They found that third-trimester blood maternal sHLA-G concentrations were significantly higher in subjects with rheumatic diseases than in controls. Moreover, cord blood sHLA-G concentrations were significantly higher in rheumatic disease than in newborns from control mothers. In addition, they found a positive correlation between maternal and fetal (i) titers of ANA autoantibodies and (ii) sHLA-G circulating levels. These data suggested that autoimmune diseases prompt a maternal and fetal immune response which favors pregnancy immune tolerance through upregulation of sHLA-G in maternal and cord blood [57].

Collectively, these novel studies confirmed the important role of HLA-G expression on EVT in the remodeling of human deciduas and in the modulation of decidual NK cell functions. Moreover, recent observations suggested that maternal and fetal genotype can affect sHLA-G levels in maternal blood and consequently the success of pregnancy. Finally, a positive correlation between polymorphisms of* HLA-G* gene and infertility, preeclampsia, and abortion has been further confirmed.

## 4. HLA-G in Autoimmune/Inflammatory Diseases

Several studies in the last years have demonstrated that HLA-G plays an important role in the control of autoimmune/inflammatory diseases, such as multiple sclerosis (MS) [86], Crohn's disease (CD) [87], psoriasis [88], pemphigus [89], celiac disease [90], systemic lupus erythematosus (SLE) [91], asthma [92], juvenile idiopathic arthritis [93], and rheumatoid arthritis (RA) [94].

Novel data have been obtained by Catamo and coworkers, who have observed that five polymorphisms (namely, -477 C>G, -369 C>A, 14 bp del/ins, 3187 A>G, and 3196 C>G) and one haplotype (TCGGTACGAAITCCCGAG) of* HLA-G* gene were significantly more frequent in celiac patients than in healthy controls and were correlated with an increased susceptibility to the disease. Moreover, they found 5 additional polymorphisms of* HLA-G* gene (14 bp I/I, 3187 G/G, 3196 G/G, and 3003 C/C genotypes and TCGGTACGAAITCCCGAG haplotype) which were associated with increased disease susceptibility only considering patients and controls presenting the DQ2.5 or DQ8 HLA-DQ celiac disease risk haplotypes. These data confirmed that* HLA-G* gene polymorphisms correlated to susceptibility to celiac disease development, suggesting that HLA-G molecule is involved in disease pathogenesis [58]. In another study, the authors observed that 10 5′URR and 3 3′UTR polymorphisms in* HLA-G* gene and two haplotypes were associated with a higher risk for RA development, while one polymorphism in the 5′URR correlated with disease activity. These data suggested a possible association between* HLA-G* gene polymorphisms and susceptibility to develop RA disease and its severity [59]. Interestingly, Mariaselvam et al. have observed that the frequency of +3187A>G HLA-G polymorphism was higher in rheumatoid factor (RF)^+^ than in RF^−^ patients, thus suggesting that this polymorphism might influence RF status [60].

In this regard, Veit and coworkers have observed that homozygosity for the +3142G allele was associated with an increased risk of RA in Brazil, and the presence of this allele in homozygosis could be responsible for a low HLA-G expression profile which favors the development of RA [61]. Moreover, they have also observed that sHLA-G is increased in RA patients with long-lasting chronic inflammation, and the percentage of patients showing specific binding of sHLA-G to LILRB1 was significantly decreased as compared to controls. Interestingly, RF^−^ patients were significantly overrepresented in the group of patients positive for LILRB1 binding. Furthermore, methotrexate treated patients had lower LILRB1 binding to sHLA-G molecules than nontreated patients. These results suggested that although increased levels of sHLA-G are observed, these molecules are not functional against inflammation, due to a low binding capacity to the receptor, thus highlighting the importance to also measure the binding capability of sHLA-G to LILRB1 [62].

Zhang et al. have observed a correlation between HLA-G 14 bp ins/del polymorphism and SLE susceptibility in Asian and Caucasian subjects [63]. In this line, Favoino et al. have analyzed serum sHLA-G and the HLA-G gene 14 bp ins/del polymorphism in patients with systemic sclerosis (SSc). They subdivided patients on the basis of sHLA-G levels in HLA-G high and low profile groups, and they detected a higher disease severity in HLA-G low than in HLA-G high group. Moreover, they detected a higher frequency of scleroderma in patients with HLA-G 14 bp del/del. These data suggested a modulatory effect of sHLA-G on SSc [64]. Similarly, Zidi and coworkers have observed a correlation between the 14 bp del/ins polymorphism and CD susceptibility in young-onset (but not in adult-onset) CD patients, concerning the genotype ins/ins. Moreover, they observed higher sHLA-G levels in CD patients than in controls, and they found that patients with 14 bp del/del and 14 bp del/ins genotypes are the high HLA-G producers. Dimers of sHLA-G were found in advanced disease stage, thus suggesting a role for sHLA-G as a prognostic marker for progressive disease in CD patients [65]. Dimers of sHLA-G have been also analyzed by Fainardi et al. in MS. They demonstrated that HLA-G dimers in cerebrospinal fluid were more frequent in MS patients than in controls and in magnetic resonance imaging (MRI) inactive than in MRI active MS patients, thus suggesting that HLA-G dimers may be implicated in termination of inflammatory response in MS [66]. Moreover, Mohammadi et al. have observed that 14 bp insertion in HLA-G could result in lower plasma HLA-G levels in patients, and they found a significant correlation of HLA-G genotype and its plasma levels with MS susceptibility, thus suggesting a role for HLA-G as a risk factor for MS [67].

de Albuquerque and coworkers have analyzed different polymorphism of 3′UTR of HLA-G gene in type 1 diabetes mellitus (T1D) patients. +3001 T allele was observed only in T1D patients, whereas the +3010 CC genotype and the UTR-3 haplotype (14 bp del/+3001C/+3003T/+3010C/+3027C/+3035C/+3142G/+3187A/+3196C), associated with low and moderate soluble HLA-G expression, respectively, were underrepresented in patients. They concluded that a decreased expression of HLA-G in pancreas should be detrimental in individuals genetically prone to produce less HLA-G [68].

Two interesting studies have evaluated the correlation between HLA-G and response to treatment.

Borghi et al. have analyzed the correlation between HLA-G 14-bp ins/del polymorphism and the response of psoriatic patients to systemic therapy (acitretin, cyclosporine, or anti-TNF-*α*). They found an increased frequency of HLA-G del allele and del/del genotype in responders only among patients treated with acitretin, and they proposed this HLA-G polymorphism as a potential marker of response to acitretin in psoriatic patients [69]. In contrast, Naidoo et al. have analyzed the relationship between HLA-G and statins treatment in patients with asthma. They demonstrated that statins upregulate mir-148b and -152, and these miRNAs can affect HLA-G expression. The binding of miRNAs to HLA-G is modulated by a SNP in the HLA-G 3′UTR that is associated with asthma risk (rs1063320). Finally, they observed that individuals with G allele of rs1063320 had reduced asthma-related exacerbations, thus suggesting that rs1063320 modifies the effect of statin benefit in asthma by modulating HLA-G expression through mir-148b and -152 [70].

We can conclude that in the last two years the ability of HLA-G to limit the progression of autoimmune/inflammatory diseases has been confirmed. In fact, sHLA-G levels and several HLA-G polymorphisms have been associated with higher susceptibility to disease or higher severity of the disease. Finally, HLA-G has been proposed also as predictive marker of response to treatment (Table 3).

## 5. HLA-G in Infectious Diseases

In the last years, the role of HLA-G in the progression of different infectious diseases has been fully described, for both microbial and viral infections [95]. Recent studies have addressed novel aspects of the role of HLA-G in bacterial infections. Han et al. have demonstrated that* Toxoplasma gondii* infection can upregulate sHLA-G release by trophoblast cells* in vitro*. Upon coculture with infected trophoblast cells, dNK cells undergo apoptosis through upregulation of caspase 3 and caspase 8. They demonstrated that apoptosis is induced by sHLA-G, since the expression of caspases is downregulated in the presence of HLA-G neutralizing antibody. The authors hypothesize that dNK cells apoptosis may contribute to the abnormal pregnancy outcomes with* T. gondii* infection [71].

Sadissou and coworkers firstly analyzed the possible association between sHLA-G and malaria or malaria related risk factors in pregnant women. They observed strong correlations between the maternal and cord plasma concentrations of sHLA-G. Moreover, high cord plasma levels of sHLA-G were independently associated with low birth weight and increased risk of* P. falciparum* infection in infancy. These results suggested a possible involvement of sHLA-G in generating immune tolerance during pregnancy-associated malaria. They concluded that sHLA-G may represent a useful marker of susceptibility to malaria in infants [72].

Bortolotti and coworkers carried out an interesting study on* Pseudomonas aeruginosa*, demonstrating that N-(3-oxododecanoyl)-l-homoserine lactone (3O-C12-HSL) produced by this bacteria is able to induce HLA-G expression in human monocytes and T cells, through p38/CREB and IL-10 induction. These data suggested that HLA-G may be a mechanism to create a protected niche for bacterial reservoir, similar to the role of HLA-G molecules during viral infections [73]. In this line, Rizzo et al. have demonstrated that sHLA-G plasmatic levels are normalized by antibiotic therapy in patients with cystic fibrosis and* Pseudomonas aeruginosa* infection, thus suggesting a systemic anti-inflammatory role. However, in the airway system, higher expression of HLA-G is associated with* P. aeruginosa* infection. Moreover, CF cell line and murine model expressed higher HLA-G molecules in the presence of* P. aeruginosa*, thus suggesting a role of HLA-G in reducing systemic inflammation, thus supporting* P. aeruginosa* infection [74].

Oliveira Souza and coworkers have detected HLA-G expression in gastric tissue samples from patients harbouring* H. pylori*. Moreover, HLA-G expression was correlated with milder colonization by* H. pylori*, milder inflammatory activity, and location of bacteria in the gastric antrum. This pilot study explored for the first time HLA-G expression in the context of* H. pylori* infection, but the role of HLA-G remains to be defined [75].

Two important studies on viral infections have been carried out. Khorrami et al. have analyzed HLA-G in patients with Hepatitis C virus (HCV) treated with combined therapy (IFN-*α*2*α* and ribavirin), to evaluate possible difference between responder and nonresponder groups. They demonstrated that HLA-G and IL-10 levels in nonresponder group were higher than in responder and controls. Additionally, HLA-G and IL-10 were higher in patients at the beginning of treatment than in healthy individuals. These findings suggested that increase of HLA-G and IL-10 in HCV infected patients might affect the response to combined therapy in HCV patients [44]. Laaribi and coworkers have analyzed HLA-G 14 bp ins/del polymorphism at the 3′ UTR of HLA-G in patients with chronic Hepatitis B virus (HBV) infection, and they found an association between the 14 bp ins/del polymorphism and an enhanced HBV activity, with high HBV DNA levels. In particular, ins/ins genotype was associated with a 2.5-fold increased risk of susceptibility to high HBV replication compared with the del/del and ins/del genotypes. These results suggest a role for HLA-G polymorphisms as potential prognostic value for disease outcome evaluation [76].

In summary, these novel studies confirmed that upregulation of HLA-G expression and/or release is an immune escape mechanism performed by virus and bacteria during infection to avoid the recognition by immune effector cells and to reduce the inflammatory response. Also in this case, a predictive role of the success of therapy has been suggested for HLA-G (Table 4).

## 6. HLA-G in Transplantation

The important role of HLA-G as a tolerogenic molecule during allograft transplantation has been extensively characterized. HLA-G expression has been related to a better acceptance of the allograft, through its ability in shaping an allogeneic immune response into tolerance [96].

Misra et al. have evaluated the impact of HLA-G allele associated with UTR-haplotype in end stage renal disease (ESRD) and acute allograft rejection (AR) cases. The authors observed an increased risk for G^*∗*^01:01:01:03, G^*∗*^01:01:02, G^*∗*^01:06, and G^*∗*^01:05:N haplotypes, while G^*∗*^01:01:01:01 and G^*∗*^01:04:01 haplotypes showed a protective effect in ESRD and AR cases. Moreover, they found (i) higher levels of soluble HLA-G5 and -G6 among ESRD cases and (ii) reduced levels of soluble HLA-G5 and increased levels of membrane-bound HLA-G1 and -G3 in AR cases. Decreased HLA-G expression was observed for G^*∗*^01:01:01:03 and G^*∗*^01:05:N haplotypes in ESRD and AR cases. These results suggested that the variation of membrane-bound and soluble HLA-G isoforms correlated to the risk for ESRD and AR. Moreover, UTR-haplotypes are involved in different HLA-G expression patterns [77].

Three recent studies have analyzed the role of HLA-G in patients who undergo lung transplantation.

White and coworkers have demonstrated that lung soluble HLA-G concentrations were directly related to the presence of type A rejection but not to lymphocytic bronchiolitis (the principal complication of lung transplantation). They found that sHLA-G concentrations in bronchoalveolar lavage but not in serum positively correlated with the number of acute rejection episodes in the first 12 months after lung transplantation and thus may represent a novel marker of rejection risk [78]. Similarly, Brugière et al. have observed that lung HLA-G expression had a protective effect on chronic lung allograft dysfunction (CLAD), thus suggesting that early expression of HLA-G in the graft was positively correlated with graft acceptance in the long term [79]. An interesting study carried out by Di Cristofaro and coworkers observed that the presence of HLA-G^*∗*^01:06 UTR2 haplotype was associated with a worse evolution of cystic fibrosis, but not of long-term posttransplant survival. In contrast, HLA-G^*∗*^01:04 UTR3 haplotype was associated with lower levels of sHLA-G at day 0 and after three months, impaired long-term survival, increased CLAD occurrence, and the production of* de novo* donor-specific antibodies after three months. This study firstly observed that different HLA-G haplotypes can deeply affect clinical outcome of lung transplantation [80].

In the view of the treatment of transplant rejection, an interesting study comes from Pankratz and coworkers. They compared the features of two subsets of regulatory cells, CD4^+^HLA-G^+^ cells and CD4^+^CD25^+^FoxP3^+^cells. They found that both cell populations display alterations in proximal-signaling pathways upon TCR stimulation and a hyperpolarization of the plasma membrane when compared to conventional CD4^+^T cells. CD4^+^HLA-G^+^ cells secreted high levels of IL-10, sHLA-G, and IL-35, while CD4^+^CD25^+^FoxP3^+^ cells expressed lower levels of these molecules and exerted their function in a contact-dependent manner via cAMP. More importantly, they demonstrated that CD4^+^HLA-G^+^ cells ameliorated graft-versus-host disease in a humanized mouse model, thus suggesting that these cells can modulate polyclonal adaptive immune responses* in vivo*, representing a promising candidate for future clinical applications not only in transplanted patients but also for the treatment of autoimmune/inflammatory diseases [81].

In conclusion, these novel findings confirmed in general the important role of HLA-G in the acceptance of allograft and in the prevention of transplant rejection. Moreover, recent studies suggested a possible clinical application of CD4^+^HLA-G^+^ regulatory T cells in the prevention of GvHD (Table 5).

## 7. Conclusions

More than 280 papers on HLA-G have been published in the last two years confirming the important role of HLA-G in modulating the function of the immune system. Moreover, novel studies have highlighted the function of HLA-G gene (and corresponding molecule) as prognostic factor for patients' clinical outcome in different pathological settings. Finally, some authors have unraveled a role for HLA-G as a predictor of the response to treatments in patients with tumors or infections. The future goal of the scientific community will be to determine and standardize the clinical applications of HLA-G analysis, in order to introduce this molecule in the routine tests which might help disease diagnosis and patients' follow-up.

## Figures and Tables

**Table 1:** Novel findings on HLA-G and tumors.

Type of tumor	Observation	Correlation with clinical outcome	PB sHLA-G	Author
Murine model	HLA-G^+^ tumors are protected from rejection	—	—	Loumagne et al. [27]
ESCC	Tumors but not adjacent tissues expressed HLA-G	Positive correlation with metastasis	Increased	Zheng et al. [28]
Rectal cancer	Tumors are HLA-G^+^, FOXP3^+^, and HLA-class I^−^	Worse OS	—	Reimers et al. [29]
Colorectal cancer	Most tumors expressed HLA-G and/or HLA-E	Worse OS	—	Guo et al. [30]
NSCLC	HLA-G is expressed in adenocarcinoma but not in squamous cell carcinoma	—	—	Yan et al. [31]
Pancreatic cancer	Most tumors are HLA-G^+^	Positive correlation with metastasis and worse OS	Increased	Xu et al. [32]
Glioma	HLA-G expression is higher in tumors with blurred boundaries	Positive correlation with invasiveness	—	Wang et al. [33]
Renal cell carcinoma	Expression of some miRNAs downregulates HLA-G expression and increased antitumor immune response	—	—	Jasinki-Bergner et al. [34]
Gastric cancer	TGF-*β* induced HLA-G expression through miR152 downregulation	—	—	Guan et al. [35]
Gastric cancer	HLA-G^+^ DC-10 cells are increased in PB of patients	Worse prognosis	Increased	Xu et al. [36]
Murine model	LILRB1^−^ NK cells are more effective against HLA-G^+^ tumors	—	—	Wu et al. [37]
Pancreatic cancer	CD3^+^ TIL are decreased in HLA-G^+^ tumors	Worse OS	—	Zhou et al. [38]
Breast cancer	Opposite prognostic value of free sHLA-G and sHLA-G in MV after chemotherapy	sHLA-G in MV positively correlated with worse prognosis; free sHLA-G positively correlated with better outcome	—	König et al. [39]
Breast cancer	Higher sHLA-G levels in patients without previous pregnancy and breastfeeding history	Worse OS	Increased	Zidi et al. [40]
DLBCL	Different prognostic value of del/del polymorphisms of HLA-G gene	Worse OS	—	Bielska et al. [41]
NSCLC	SNP in *HLA-G* and *LILRB1* genes and expression of HLA-G and LILRB1 are related to clinical outcome	Positive correlation to high risk of tumor and tumor stage	—	Wiśniewski et al. [42]
Prostate cancer	Polymorphisms in 3′ UTR of *HLA-G* gene are related to clinical outcome	Positive correlation to higher susceptibility	—	Zambra et al. [43]
Gastric adenocarcinoma	Higher frequency of some polymorphisms of *HLA-G* gene in healthy controls than in patients	—	—	Khorrami et al. [44]
CLL	14 bp polymorphism in 3′ UTR of *HLA-G* gene is related to clinical outcome	Worse OS	—	Rizzo et al. [45]

**Table 2:** Novel findings on HLA-G and pregnancy.

Observation	Correlation with outcome of pregnancy	Author
HLA-G^+^ EVT but not HLA-G^−^ VT have immune activating potential and induce Tregs	—	Tilburgs et al. [13]
dNK acquired HLA-G through interaction with EVT and can be reactivated by cytokine stimulation	—	Tilburgs et al. [46]
High HLA-G levels in placental bed correlated with high expression of KIR2DL4 and LILRB1 on dNK cells	—	Djurisic et al. [47]
Enhancer L on *HLA-G* gene is controlled by CEBP and GATA that are also essential for placentation	—	Ferreira et al. [48]
sHLA-G is higher in maternal blood at GW20 than at term and higher in maternal blood than in umbilical blood	—	Klitkou et al. [49]
Higher 14 bp alleles in fetus are related to higher sHLA-G in maternal blood at term	—	Dahl et al. [50]
sHLA-G in seminal plasma is related to 14 bp ins/del genotype and can predict the success of ART	Positive correlation with the success of ART	Dahl et al. [51]
Higher frequency of 14 bp ins alleles in *HLA-G* gene is related to the outcome of pregnancy	Positive correlation with RIF	Lashley et al. [52]
Higher sHLA-G and frequency of KIR2DL4^+^ NK cells in uterine flushing samples from secondary infertile women than in primary infertile woman	Negative correlation with infertility	Rizzo et al. [53]
SNP in 3′ UTR of *HLA-G* gene is related to outcome of pregnancy	Positive correlation with high risk of preeclampsia	Quach et al. [54]
SNP in 5′ URR of *HLA-G* gene is related to outcome of pregnancy	Positive correlation with RSA	Agrawal et al. [55]
Lower sHLA-G and PAPP-A in women with GDM at third trimester	—	Beneventi et al. [56]
Higher sHLA-G in women with preexisting rheumatic disease at third trimester	Positive correlation with the success of pregnancy	Beneventi et al. [57]

**Table 3:** Novel findings on HLA-G in autoimmune/inflammatory disease.

Type of disease	Observation	Correlation with clinical outcome	Author
CD	Several polymorphisms and one haplotype of *HLA-G* gene are more frequent in patients than in controls	Positive correlation with susceptibility	Catamo et al. [58]
RA	Several polymorphisms and two haplotypes of *HLA-G* gene are more frequent in patients than in controls	Positive correlation with susceptibility	Catamo et al. [59]
RA	Two polymorphisms of *HLA-G* gene are more frequent in RF^+^ than in RF^−^ patients	Positive correlation with disease severity	Mariaselvam et al. [60]
RA	Homozygosis of *3142G* allele is related to lower sHLA-G levels	Positive correlation with high risk of disease	Veit et al. [61]
RA	Higher sHLA-G levels in patients with chronic disease and higher LILRB1 binding in RF^−^ patients	Negative correlation with disease severity	Veit et al. [62]
SLE	*HLA-G *14 bp ins/del polymorphisms are associated with susceptibility	Positive correlation with susceptibility	Zhang et al. [63]
SSc	Lower levels of sHLA-G in patients than in controls	Negative correlation with disease severity	Favoino et al. [64]
CD	Higher levels of sHLA-G in patients than in controls	Positive correlation with susceptibility	Zidi et al. [65]
MS	Higher sHLA-G dimers in MS than in controls and in MRI^−^ than in MRI^+^	Positive correlation with termination of inflammatory response	Fainardi et al. [66]
MS	14 bp ins in *HLA-G* gene is related to lower sHLA-G levels	Positive correlation with high risk of disease	Mohammadi et al. [67]
T1D	Lower sHLA-G levels in pancreas are detrimental	Negative correlation with disease severity	de Albuquerque et al. [68]
Psoriasis	14 bp del/del genotype of *HLA-G* gene is more frequent in patients responding to therapy	Positive correlation with response to treatment	Borghi et al. [69]
Asthma	SNPs in 3′ UTR of HLA-G gene modulate the binding of mIRNAs	Positive correlation with high risk of disease	Naidoo et al. [70]

**Table 4:** Novel findings on HLA-G and infectious diseases.

Type of disease	Observation	Correlation with clinical outcome	Author
Toxoplasmosis	Toxoplasma increased the release of sHLA-G by trophoblast inducing apoptosis of dNK cells	Positive correlation with abnormal pregnancy	Han et al. [71]
Malaria	Higher sHLA-G levels in cord blood are related to low weight at birth and clinical outcome	Positive correlation with high risk of infection in infancy	Sadissou et al. [72]
*Pseudomonas aeruginosa*	*P. aeruginosa* induced HLA-G expression in monocytes and T cells protecting from immune response	—	Bortolotti et al. [73]
*Pseudomonas aeruginosa*	sHLA-G levels are decreased during antibiotic therapy in patients with cystic fibrosis	Negative correlation with inflammation	Rizzo et al. [74]
*Helicobacter pylori*	HLA-G expression correlated with milder colonization and milder inflammation	Negative correlation with inflammation	Oliveira Souza et al. [75]
HCV	Higher levels of sHLA-G and IL-10 in patients nonresponding to therapy with IFN	Negative correlation with response to therapy	Khorrami et al. [44]
HBV	Patients with *14 bp ins/ins* genotype have higher levels of HBV activity and HBV DNA copies	Positive correlation with worse clinical outcome	Laaribi et al. [76]

**Table 5:** Novel findings on HLA-G and transplantation.

Type of transplant	Observation	Correlation with clinical outcome	Author
Kidney	Higher HLA-G5/G6 levels in ESRD and lower levels of HLA-G5 in acute rejection	Negative correlation of HLA-G5 with rejection	Misra et al. [77]
Lung	Higher levels of sHLA-G in bronchoalveolar lavage from patients with acute rejection	Positive correlation with high risk of rejection	White et al. [78]
Lung	Expression of HLA-G in lung protected from CLAD and associated with graft acceptance	Positive correlation with graft acceptance	Brugière et al. [79]
Lung	Two haplotypes of HLA-G gene are associated with lower levels of sHLA-G	Positive correlation with worse long term survival	Di Cristofaro et al. [80]
Murine model	CD4^+^HLA-G^+^ regulatory T cells produce IL-10, sHLA-G, and IL-35 and ameliorated GvHD	—	Pankratz et al. [81]
